# “I can guess the month … but beyond that, I can’t tell” an exploratory qualitative study of health care provider perspectives on gestational age estimation in Rajasthan, India

**DOI:** 10.1186/s12884-020-03201-6

**Published:** 2020-09-11

**Authors:** K. Scott, S. Gupta, E. Williams, M. Arthur, U. V. Somayajulu, L. Noguchi

**Affiliations:** 1 USAID’s Maternal and Child Survival Program/Johns Hopkins Bloomberg School of Public Health, Baltimore, USA; 2 USAID’s Maternal and Child Survival Program/Jhpiego, Baltimore, USA; 3 USAID’s Maternal and Child Survival Program/USAID, Washington, D.C., USA; 4 Sigma, Delhi, India

**Keywords:** Gestational age estimation, Neonatal health, Preterm birth, India, Qualitative, Client-provider relationships, Quality of care, Antenatal care, Ultrasound

## Abstract

**Background:**

Accurately estimating gestational age is essential to the provision of time-sensitive maternal and neonatal interventions, including lifesaving measures for imminent preterm birth and trimester-specific health messaging.

**Methods:**

We explored healthcare provider perspectives on gestational age estimation in the state of Rajasthan, India, including the methods they use (last menstrual period [LMP] dating, ultrasound, or fundal height measurement); barriers to making accurate estimates; how gestational age estimates are documented and used for clinical decision-making; and what could help improve the accuracy and use of these estimates. We interviewed 20 frontline healthcare providers and 10 key informants. Thematic network analysis guided our coding and synthesis of findings.

**Results:**

Health care providers reported that they determined gestational age using some combination of LMP, fundal height, and ultrasound. Their description of their practices showed a lack of standard protocol, varying levels of confidence in their capacity to make accurate estimates, and differing strategies for managing inconsistencies between estimates derived from different methods. Many frontline healthcare providers valued gestational age estimation more to help women prepare for childbirth than as a tool for clinical decision making. Feedback on accuracy was rare. The providers sampled could not offer ultrasound directly, and instead could only refer women to ultrasound at higher level facilities, and usually only in the second or third trimesters because of late antenatal care-seeking. Low recall among pregnant women limited the accuracy of LMP. Fundal height was heavily relied upon, despite its lack of precision.

**Conclusion:**

The accuracy of gestational age estimates is influenced by factors at four levels: 1. health system (protocols to guide frontline workers, interventions that make use of gestational age, work environment, and equipment); 2. healthcare provider (technical understanding of and capacity to apply the gestational age estimation methods, communication and rapport with clients, and value assessment of gestational age); 3. client (time of first antenatal care, migration status, language, education, cognitive approach to recalling dates, and experience with biomedical services); and, 4. the inherent limitations and ease of application of the methods themselves.

## Plain language summary

Knowing the age of a fetus helps doctors and nurses give appropriate advice and make important healthcare decisions. Yet in many low-resource settings, doctors and nurses struggle to accurately estimate fetal age. This study asked 30 nurses, doctors, and policy experts in India about how fetal age is estimated and used in the government sector. We found that healthcare providers relied primarily on calculating fetal age based on the pregnant woman’s last menstrual period (LMP) or by measuring fundal height (size of the pregnant belly), but they struggled with low client recall of LMP and reported using their hands rather than measuring tapes for fundal height measurement. Early ultrasound, which is the gold standard for estimating fetal age, was rarely used because pregnant women sought healthcare too late, found ultrasounds difficult to access, and considered them a low priority. Strategies used for estimating fetal age varied widely from provider to provider. Many doctors and nurses were overconfident about their capacity to make accurate estimates, and only a minority recognized the inherent limitations to the estimation methods. We present a framework of factors at the health system, healthcare provider, client, and method/tool levels that must be improved to make fetal age estimation and use more successful. Strategies include increasing the health system’s capacity to actually use these estimates to help women and infants, simplifying documentation, improving healthcare provider training and supervision (including on client-provider communication), encouraging newly pregnant women to visit healthcare workers as early as possible, increasing ultrasound accessibility, and improving women and health workers awareness of the value of accurate estimates.

## Background

13.7 million preterm births (births prior to 37 weeks’ gestation) occur annually in low- and middle-income countries (LMICs) – over 90% of the global burden [1]. Preterm birth complications are the leading cause of mortality among children under five years [2]. Of the 2.7 million annual neonatal deaths (occurring in the first 27 days), 0.94 million (35%) are attributable to conditions related to preterm birth [2] [3]. In India, approximately 3.5 million infants are born too early each year [1] and preterm birth-related conditions are the cause of 0.3 million deaths among Indian children under five [2]. Conditions related to preterm birth cause 43% of all neonatal mortality and 27% of all under five mortality in India [2].

Knowing the gestational age of a fetus is vital to the provision of antenatal corticosteroids to women at risk of preterm delivery, which could save 370,000 lives each year [3]. While the administration of antenatal corticosteroids is recommended for women at risk of preterm birth from 24 weeks to 34 weeks, administration after 34 weeks’ gestation brings risk of harm to mature fetuses exposed to corticosteroid in-utero that may outweigh the benefits [4]. The World Health Organization thus recommends that: “antenatal corticosteroid should not be routinely administered in situations where the gestational age cannot be confirmed” [4].

Accurate gestational age assessment also enables the timely initiation of appropriate care for a preterm infant. Furthermore, many aspects of maternal healthcare rely on gestational age estimates, including mobile phone-based health messaging programs that provide stage-based pregnancy information to millions of women in LMICs [5, 6].

In high-resource settings, early pregnancy ultrasound-based dating is standard practice and considered the most accurate method of gestational age estimation. The gold standard is ultrasound prior to 14 weeks gestation, while estimates prior to 22 weeks are considered adequate [7]. Ultrasound-based dating during the first trimester often uses crown–rump length to estimate gestational age, since there is a linear relationship between this measurement and gestation age during early pregnancy [8]. Later in pregnancy, gestational age can be estimated, albeit with lower accuracy, through ultrasound measurement of combinations of biparietal diameter, head circumference, abdominal circumference and femur (diaphysis) length [9]. Menstrual-based dating is also widely used, and calculates the estimated date of full term delivery (40 weeks’ gestation) as 280 days from the first day of a woman’s last menstrual period (LMP) [10], but is only accurate among women who menstruated regularly and can recall their LMP.

In low-resource settings, gestational age estimates are often inaccurate [11], making it difficult for health care providers to take appropriate clinical decisions. Little is known about how health care providers in these settings determine estimated date of delivery, their use of and confidence in various gestational age estimation methods, and their use of gestational age estimates for identifying and managing induction of labour, imminent pre-term birth, post-term and complicated pregnancies, and preterm neonates. Systems for documenting and using gestational age estimates throughout pregnancy are also under-researched, despite their importance in enabling gestational age estimates to inform the provision of time sensitive interventions.

Measuring fundal height is one low-cost approach used in low-resource settings [12]; however, it only generates approximate gestational age estimates, is dependent on provider skill, and does not differentiate between growth restriction and lower gestational age – a particularly important issue in regions with endemic malaria, as malaria is strongly associated with intrauterine growth restriction [13]. Even when multiple (up to seven) fundal height measurements are entered into population-specific models accounting for fundal height and slope (gradient), researchers have found this gestational age estimation method produces too great a range to enable the classification of prematurity [14].

This paper presents qualitative findings on the practice of gestational age estimation, documentation, and utilization for clinical care of pregnant women in low-resource health facilities in Rajasthan, India. The aim of this research is to document current systems and protocols in order to identify facilitators and barriers to accurate gestational age estimation, appropriate documentation of gestational age estimates, and correct use of gestational age estimates to provide appropriate gestational age-specific interventions during pregnancy and delivery.

## Methods

### Study setting

Research took place in Rajasthan, a state in north-western India with a population of 78 million. Rajasthan was selected in consultation with the Ministry of Health and Family Welfare, as part of its focus on improving maternal and newborn health care in Empowered Action Group states. While 91% of households had some (often limited and irregular) access to electricity, only 45% had improved sanitation [15]. Male literacy was 85%, female literacy was 57% and only 25% of women had 10 or more years of schooling [15]. In 2015, 63% of pregnant women received antenatal care (ANC) in the first trimester and 38% received the recommended four ANC visits [15]. The institutional birth rate was 84, and 64% of institutional births were in a public facility [15]. The total fertility rate was 2.4 children per woman and infant mortality was 41 per 1000 live births [15]. Rajasthan continues to experience endemic malaria [16].

### Data collection

The study consisted of 30 in-depth interviews with frontline ANC providers (*n* = 10), intrapartum care (IP) providers (*n* = 10) and expert key informants (KI) in the government (public) health sector (*n* = 10) (Table 1). Our sampling was guided by the principle of thematic saturation for the healthcare providers [17], while key informants were sampled based on access and availability across a pre-set range of profiles. We note however that the ANC and IP classifications are not rigid and merely indicate the focus of each respondent’s work because many ANC providers also conduct deliveries and all IP providers at least occasionally provide ANC.

**Table 1:** Qualitative respondents and their profiles

Respondent group	Respondent title	Profile	Male	Female	Row total
Antenatal care provider	Auxiliary nurse midwife (ANM)	Female maternal and child health worker with 1.5 years training (6 months midwifery focused)	0	9	9
	Lady health visitor	ANM with 5+ years’ experience and 6 months additional upgrade training	0	1	1
Intrapartum care provider	Staff nurse	Registered nurse with 3 (General Nurse Midwife) or 4 (BSc Nursing) years training (~ 6 months midwifery focused)	1	3	4
	Medical officer (MO)	Bachelor of Medicine, Bachelor of Surgery (MBBS) doctor, 5.5 years training	5	1	6
Key informant	Medical officer: senior, principle, or in-charge	MBBS doctor in a supervisory role at district level hospital	1	3	4
	Technical coordinator	Ministry of Health and Family Welfare (MoHFW) program and technical expert staff	1	0	1
	Nursing college principal or vice principal	Nurse now working in the education sector	0	3	3
	Reproductive and child health officer	District level government health program officer	1	0	1
	Maternal and child health head	District level government health program officer	1	0	1
		Column total	10	20	30

Respondents were located in rural health sub-centers (*n* = 8), rural primary health centers (*n* = 8), district hospitals (*n* = 8), nursing colleges (*n* = 3) and ministry or department of health offices (*n* = 3). ANC providers, which included auxiliary nurse midwives (ANMs) (*n* = 7), a staff nurse (*n* = 1) and a medical officer (*n* = 1), are the first point of contact in the government health system for pregnant women. They determine the initial gestational age estimate and generate a woman’s pregnancy-related documents. Intrapartum care providers, which included ANMs (*n* = 2), staff nurses (*n* = 3), a lady health visitor (*n* = 1) and medical officers (*n* = 4), make use of gestational age estimates and ANC documents to provide care throughout labour and delivery; many of them also conduct some antenatal care. They also assess and refer complex cases to higher level facilities. Key informants, which included medical officers in supervisory and tertiary care roles (*n* = 4), government health program coordinators and officers (*n* = 3), and teachers in nursing colleges (*n* = 3), provided macro level perspectives on gestational age estimation as educators, experienced and senior healthcare providers, or as policymakers working in maternal and child health.

Data were collected by three qualitative researchers (two female and one male) after a two-day training in Delhi; all three had experience in public health qualitative research but none were trained in nursing or paramedic subjects. Training included orientation to the clinical components of gestational age estimation. Data collection took place between January and April 2018 in five districts of Rajasthan state (Udaipur (*n* = 6), Bundi (*n* = 3), Pratapgarh (*n* = 5), Bharatpur (*n* = 10), Swaimadhopur (*n* = 2)) and the national capital, Delhi (*n* = 4). Interviews ranged from 38 to 67 min and were conducted in Hindi then translated and transcribed into English.

The interviews explored a range of domains, including: the respondent’s work environment (client load, relationships, supervision); typical gestational age estimation methods used and their perceived accuracy; issues around late ANC care-seeking; challenges to determining accurate gestational age and strategies to achieve accurate gestational age estimates; how gestational age estimates are used during antenatal and intrapartum care, including the identification and management of pre-term labour and infants; gestational age-related training received; gestational age-documentation practices; and recommendations for improving gestational age estimation, documentation, and use (the ANC interview guide is provided in Annexure 1).

### Data analysis

**Fig. 1:**
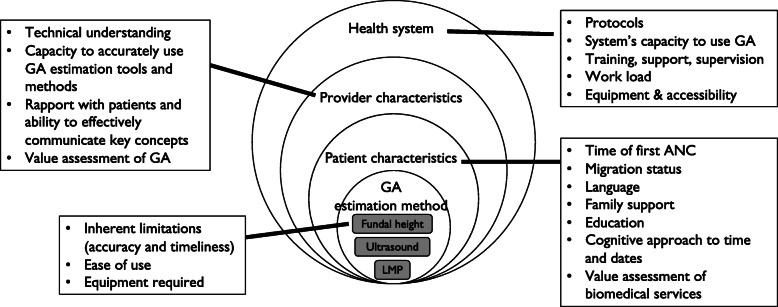
Factors influencing gestational age estimation, documentation, and use

Analysis began with an initial review of five transcripts and the development of a codebook. The codebook consisted of 176 sub-codes developed from a priori areas of interest as well as topics emerging from the data. These sub-codes were grouped into 34 main codes based on the a priori interests of the study. For example, we had an a priori interest in reasons for late ANC care-seeking among pregnant women, so when respondents spoke about migrant workers sometimes not coming to the health centre until the very end of their pregnancy we created the sub-code “migrant labourers, those who leave the village” and grouped it under the main code “late ANC/low care-seeking.” Transcripts were coded in the qualitative data management software Dedoose (Los Angeles, CA). Code reports were generated by listing all text from the transcripts that were “tagged” with a specific sub-code, along with characteristics of the respondents who made each statement. The code outputs were read for each topic and salient overarching themes were identified guided by thematic network analysis [18]. Each theme is described in the findings section below, and example quotations from the transcripts are provided. Finally, we synthesized the thematic findings into a framework that summarizes the factors influencing gestational age estimation, documentation and use (Fig. 1, presented in the discussion).

### Ethics

Ethical approval was received from the Johns Hopkins Bloomberg School of Public Health Institutional Review Board and Sigma, India. All respondents provided written informed consent before participation.

## Results

We first provide an overall picture of the gestational age estimation situation in rural Rajasthan, including details on the use of the specific estimation methods (LMP, fundal height and ultrasound). We then report findings on the documentation and use of gestational age estimates in the government health sector. We finish with provider comments on potential avenues to improving gestational age estimate accuracy. Respondents are identified by a code indicating whether they primarily provided intrapartum (IP) or ANC as well as their serial number.

### Lack of clear and consistent protocol for gestational age estimation

Providers discussed using a combination of LMP, fundal height, and ultrasound to determine gestational age. However, they also had widely varying assessments of the relative use and accuracy of the different methods. Thirteen of the 20 respondents involved in day to day ANC or intrapartum care in the government (public) sector described estimating gestational age through assessing LMP and fundal height but placed different weights on the relative importance and accuracy of the two. Six respondents (IP01, M, MO; ANC10, M, staff nurse; ANC08, F, ANM; IP02, F, staff nurse; IP03, F, lady health visitor; IP04, F, staff nurse) generally relied on LMP and fundal height equally.

[We calculate gestational age] by checking the abdomen through fundal height, and according to the time mentioned by the women we use LMP. (ANC10, M, staff nurse)

Four providers (ANC03, F, ANM; ANC05, F, ANM; ANC07, F, ANM; IP06, F, MO) relied on LMP but checked fundal height as a secondary confirmation.

We ask her the LMP and accordingly we note down her EDD [estimated date of delivery]. And we tell her the date. After that once again we do the fundal height test to be sure or in case that date has elapsed. (ANC07, F, ANM)

One provider (IP10, M, MO) said that LMP, fundal height and ultrasound were used equally to determine gestational age, although ultrasound was most accurate, and two providers (IP08, F, ANM; ANC04, F, ANM) described using fundal height only when a woman could not recall LMP:

First we ask the LMP date during the ANC, if they tell us then we calculate the EDD. However, if they are not able to tell their LMP then we calculate by fundal height. (IP08, F, ANM)

Among the seven respondents who did not describe primarily using both LMP and fundal height, five spoke only of using LMP, or heavily emphasized LMP (ANC01, M, MO; ANC02, F, ANM; ANC06, F, ANM; IP07, F, ANM; IP09, F, staff nurse) and two said they relied solely on fundal height (ANC09, M, MO; IP05, M, MO). We asked providers what they would do if LMP and fundal height indicated different estimated gestational ages. Most said they would ask the woman to have an ultrasound, while also explaining that few women actually go to have ultrasound examinations done (discussed in the sub-section on ultrasound, below).

Many healthcare providers expressed high confidence in the accuracy of their methods; only a minority reflected on the limitations inherent to these methods:

[Back when I conducted deliveries] the EDD was almost correct; there was only a difference of 1-2 days. (IP07, F, ANM)

In case the woman has come for her delivery, we will get to know how many months pregnant she is. With the kind of experience we have, we are able to make an assumption as to when the women had her last period and how many months pregnant the woman is. (IP04, F, staff nurse)

We give them almost perfect date after abdominal exam. (IP03, F, lady health visitor)

[ … ] we calculate their LMP and it comes accurate and only difference comes of five days or ten days. (ANC02, F, ANM)

There is no 100 percent accuracy. There can only be an estimate; the date of delivery can vary from 7 to 10 days before or 5 days after the expected date. We can just make an assumption that the delivery is expected to happen near a calculated date. (IP10, M, MO)

We get the exact [EDD] from the fundal height. And we can’t tell it accurately through LMP. Mostly have come around one month difference or 4 month, 4 week difference comes. (ANC09, M, MO)

It is noteworthy that many providers spoke about gestational age in terms of months, rather than weeks – potentially indicating acceptance or recognition of low precision.

### Last menstrual period: preferred method but difficult to assess

When probed on their preferred method for assessing gestational age, almost all providers explained that they would ideally use LMP but resorted to using fundal height and sometimes ultrasound because women could not recall their LMP. Providers explained that most of their female clients in rural Rajasthan understood time in relation to harvests and festivals, rather than months and days.

We ask LMP. Ladies were not so much aware about LMP [ … ] They said that it is happens at the harvest time, when there was no moon, full moon was there. Accordingly, we have to calculate LMP. We have to face many problems like in which month and date the moon was? When was the harvest? We have to calculate accordingly because they are uneducated people and will not understand. Accordingly, we calculate LMP. That is why differences occurs in LMP. (ANC03, F, ANM)

While some providers felt quite confident that they could derive an accurate LMP from harvest events, festivals or moon cycles (e.g., ANC03, F, ANM), others lamented moving from rough time periods to a specific estimate, noting a limitation in accuracy (e.g., ANC07, F, ANM). Overall, we found a very wide range in provider estimates of the percentage of women who could accurately recall LMP. Despite working with similar client populations, some providers said that only 10% of women recall LMP, while others said 20, 50 80% or almost all (98%) women recall it. Providers found moving from LMP to EDD straightforward; they easily recalled the formula of 9 months plus 7 days, and noted that all ANC registers had a calendar or chart to help them calculate and that during data entry the EDD was generated by the computer from the LMP.

Interviewees identified the following client profiles as most unable to report a precise LMP: Uneducated women, tribal women (*adivasis*, who were considered very shy about menstruation), Muslims (Mewat/Meo), people with many children and frequent pregnancies (because, according to some providers, they are careless and uneducated), older women (who generally have low education and are also shy about the fact that they remain sexually active), newlywed women (also very shy about anything to do with sex or menstruation), and women who speak a regional dialect rather than Hindi (because they struggle to communicate with their Hindi-speaking healthcare providers).

While some providers spoke of working with their clients to move from recalling menstruation in relation to festivals or the lunar cycle to identifying a date on the Gregorian calendar, most providers could not describe any way of putting women at ease or asking follow up questions to try to access a more accurate estimate of LMP. Two providers (ANC09, M, MO, ANC04, F, ANM) and one teacher in a nursing college (KI02, F, principal of nursing college) mentioned asking the woman’s mother-in-law, sister-in-law, or community health worker (ASHA or anganwadi) to help recall her LMP. When asked about strategies to get accurate LMP and reasons why LMP may be inaccurate, no providers noted that they confirmed that the woman understood that they were seeking the first day of her LMP rather than middle or last day, none confirmed that women were recalling LMP and not their first missed period and only one provider (KI07, F, MO, district hospital) mentioned checking that the respondent had a regular period.

A senior medical officer (KI07) explained several ways through which she worked with her clients to access a best-possible LMP:

We try to talk to her in her language. Like we ask her, “When was the time you used the cloth last time?” Then she tells the time in accordance to the full moon, half-moon or no moon; we ask her the details in the way she can answer. Then we calculate the date. We ask her if her menses were regular. We ask her the duration of her menses. (KI07)

Very few providers noted reasons why LMP may not be an appropriate gestational age assessment mechanism for all women. Being unable to report LMP was attributed to women’s inability to recall this information, with few providers noting that some women may have irregular periods (KI07, senior medical officer), mistake spotting for menstruation (KI01, technical coordinator, MoHFW), be on a birth control that stops menstruation (ANC08, F, ANM, ANC07, F, ANM), get pregnant after a prior pregnancy and before resuming menstruation, or think that missed periods are due to menopause rather than pregnancy (ANC07, F, ANM).

### Fundal height: an assessment in the healthcare provider’s hands

In a context of low confidence in LMP and poor ultrasound accessibility, fundal height offered one form of gestational age estimation that was always available to healthcare providers. Fundal height was frequently – but not universally – assessed during each antenatal care checkup after the first trimester. While some providers gave detailed descriptions of their method of calculating gestational age from fundal height and were confident in their capacity (ANC03, F, ANM; ANC09, M, MO), others were vague or unsure (KI10, M, MCH head, district hospital). The use of hand- or finger-based estimates rather than measuring tapes was commonly reported. No providers discussed client characteristics that could influence fundal height, such as the woman’s weight.

R: If the fetus or fundus is near the navel then the woman is 7 months pregnant. If it’s below the navel then the fetus is 5 months old. [I measure] by the palm of my hands. So how much above or below the fetus is; we get to know the gestational age of the fetus. If it is hollow underneath the navel and the fetus comes down at the lower abdomen, then it is the ninth month. But the fundal height method does not confirm the exact date of delivery like it does in the LMP method. I can’t tell the fixed days by that [fundal height] method. I can guess the months but beyond that, whether it is the starting of 5 or 6th month or end; I can’t tell that.

I: Have you ever performed it on anyone?

R: I have done it but not much. Only when they are not able to tell their LMP. (IP08, F, ANM)

Providers also assessed fundal height to track fetal growth, noting that a small fundus could indicate growth restriction while a large fundus could indicate twins or other issues. The fact that these dual uses of fundal height assessment were at odds was only discussed by one respondent (ANC10, M, staff nurse).

Providers reported that women were generally comfortable with the physical examination of their abdomen, noting that they “are quite keen on getting it done” (ANC06, F, ANM) because it reassures them that the fetus is developing well and that women “feel satisfied that they got checked” (ANC08, F, ANM). Two respondents (ANC09, M, MO and ANC10, M, staff nurse) noted gender considerations, specifying that women prefer a female healthcare provider or that male doctors are sure to keep a female attendant in the room while checking fundal height.

### Ultrasound: inaccessible and few noted importance of early examination

Provider recommendations on when ultrasound is required, how often, and whether it is needed vary widely. Most ANC providers primarily recommended ultrasound during pregnancy in cases of bleeding, pain, a history of miscarriage, or suspected complications. Some providers said it should be routinely performed in the second or third trimester to check for abnormalities. Most, but not all, providers reported that they sent women for ultrasound examinations if they could not recall LMP or when LMP and fundal height suggested different gestational age estimates.

Sometimes few of them forget their LMP; through ultrasound we can find gestational age. (ANC05, F, ANM)

Because some ladies are not able to tell their LMP date we advise them to get ultrasound done so that they will get to know exactly about the baby. (ANC02, F, ANM)

Women get their ultrasound done on their own. [ … ] Everyone does not get their ultrasound done. We only suggest getting an ultrasound done when we see there are complications. I don’t suggest everyone for it. Many of them are not in a position to get it done. (ANC07, F, ANM)

While three providers (IP06, F, MO; IP03, F, lady health visitor, IP01, M, MO) noted that ultrasound during the first trimester enabled accurate gestational age estimation, two respondents (KI01, technical coordinator, MoHFW and ANC06, F, ANM) said that ultrasound should not be done during the first trimester. Providers noted that women were increasingly coming for their first ANC checkup in the first trimester, but that late ANC remained an issue for women living in conservative households (particularly those practicing *purdah,* the physical segregation and veiling of women), migrants, and older women who already have several children.

While asking LMP and measuring fundal height could be done by the providers themselves, most could only suggest that women go for ultrasound examinations, since ultrasound facilities were only available in higher level facilities or private clinics. Most providers said that few women had any ultrasound done during pregnancy; however estimates of the percentage of women having ultrasound ranged from 5% (IP05, M, MO) to 30% (IP10, M, MO) to 90% (IP04, F, staff nurse). Having an ultrasound requires spending money in the private sector or waiting for many hours in the public sector. Additional reasons for low uptake included that women were discouraged by older family members, could not find anyone to accompany them (and were not able to travel alone due to social norms); did not want to or could not leave the house; could not afford the cost including for transportation; and a prevailing sense that ultrasound would be a waste of time and money in normal uncomplicated pregnancies.

Nobody wants to get the ultrasound done over here in the village. Even if they want to they can’t because they don’t have enough money. The elderly people say, “No point getting it done.” (IP07, F, ANM)

Confidence in the capacity of ultrasound to enable accurate gestational age estimation varied, with some (ANC06, F, ANM, ANC09, M, MO, IP05, M, MO and ANC03, F, ANM) saying it is highly accurate and/or the preferred gestational age estimation method and others (ANC07, F, ANM; IP09, F, staff nurse; IP04, F, staff nurse) considering ultrasound to be inaccurate. A nursing college teacher was unsure: “I’m quite unsure whether the ultrasound is that much reliable or not” (KI02, F, principal of nursing college).

Ultrasound is very useful to us. Estimation of EDD by ultrasound is accurate. [ … ] [We use ultrasound] after the completion of 3 months. And in sixth month if a woman faces any problem [ … ] (IP05, M, MO)

But mostly the EDD that is proposed by the ultrasound is inaccurate. It is only one or two percent where the dates are correct. At times the delivery happens 15 days before the date proposed by ultrasound; at times it is 10 days post the date proposed. (ANC07, F, ANM)

### Challenges in the work environment and non-specific understandings of the clinical benefit of accurate gestational age

Providers noted their high workload and overcrowding in health facilities as a barrier to accurate record keeping and proper gestational age estimation: “Due to workload pressure sometime it feels difficult to complete it [the ANC register]” (ANC04, F, ANM). They reported initial or refresher medical education on gestational age estimation, but none had received training on how to handle instances when gestational age estimates from different sources did not match or on when gestational age estimates should be revised. Multiple records were created and maintained (e.g., ANC book which is kept with the ANM, an ANC registration form which is sent to the higher level health facility, a Mamta card which the pregnant woman keeps with her, an Anganwadi record), which created a high documentation burden.

Some providers were frustrated by their “uneducated” women (usually in reference to Muslim and tribal women) who did not abide by medical advice, particularly for family planning and immunization. Some providers struggled to communicate with their female clients because the women spoke a regional dialect. Both male and female healthcare providers reported that some women were too “shy” and were difficult to communicate with about LMP or sexual activity.

Respondents noted that women often forgot their ANC cards and ultrasound reports for future ANC visits and at time of delivery because they were in a hurry, and labour pain made it difficult to think of anything else, they were not aware that the records were important and women’s in-laws and husband do not “take care” about these documents (IP06, F, MO). Intrapartum care providers reported that although they might check LMP in the client’s ANC card, if the woman brought it with her, ANC documentation was primarily used for record keeping rather than for clinical decision making at the time of delivery. Instead, providers conducted their own examinations at time of delivery and asked the woman again for her LMP and EDD.

At last moment documentation is not necessary. During delivery the patient is tackled first, her condition is examined, we check whether labour is absent or we have to induce labour. (KI01, technical coordinator, MoHFW)

Even if the ANM gives them the monthly record card, still the patient will have to remember their dates for the period and note them down or tell the ANM or us. (IP10, M, MO)

Interviewer: What is the purpose of those documents?

Respondent: They are used for maintaining records. (ANC10, M, staff nurse)

Intrapartum care providers report that women’s ANC records tended to be incomplete. They attributed gaps in records to: pregnant women coming irregularly for ANC; women not knowing or having key data, such as lacking a marriage certificate, Bhamashah (health insurance) number, or bank account; communication barriers (shyness, linguistic issues); high health provider workload; and frontline providers making general estimates of LMP, blood pressure and other parameters.

We asked antenatal and intrapartum care providers about the value of gestational age estimation. Only four of 20 spontaneously noted that gestational age could be a criterion for clinical decision making around the time of labour. Most providers focused on gestational age as generally useful for enabling women and their families to prepare for the baby’s birth, and for tracking fetal growth. One mentioned that gestational age enabled them to tailor advice based on the trimester (e.g., when to take iron and calcium supplements, to rest and come for frequent checkups in the final month) (ANC09, M, MO), three mentioned that gestational age was used to intervene in cases of post-term pregnancy (ANC05, F, ANM, IP06, F, MO; IP09, F, staff nurse), one mentioned that it enabled them to know that a baby was preterm (IP09, F, staff nurse).

On probing, all intrapartum care providers reported the use of corticosteroid drugs (dexamethasone) in cases of immanent pre-term birth. When asked how they identified instances of pre-term labour, they mentioned using some or all of the following, depending on what information was available: the pregnant woman’s telling them that she is only seven or 8 months pregnant but experiencing labour pain, the LMP and EDD written on the client’s chart, the size of her abdomen, and any ultrasound reports provided.

They will see the Mamta Card, LMP, EDD and the ultrasound report. They will ask the patient how many months pregnant she is. After checking all this they will know [if it is a case of pre-term labour]. (IP04, F, staff nurse)

When the patient is in labour and the EDD and fundal height are noted to be less, then we get to know about it [preterm labour]. Every woman gets to know when it has only been 8 months or 7 months. (IP01, M, MO)

Interviewer: How would you discover that it is a preterm birth?

R: Either by the report or by looking at her stomach as the stomach will be very small as compared to those of full term pregnancies. Also by checking the LMP, EDD and ultrasound reports, we can tell.

I: What documents do you check for their EDD?

R: Mamta card and ultrasound report. And the woman also tells that she is so and so months pregnant or that her EDD was that of a future date but she is having the pain now.

I: Is there any other way to find out?

R: The woman will be considerably weaker, she will be anemic. (IP04, F, staff nurse)

### Ideas for improving gestational age estimation and documentation

When asked how gestational age estimation could be improved, providers primarily focused on educating women about the importance of remembering LMP and suggested that community health workers (called ASHA sahyoginis or anganwadi workers) could conduct additional outreach to encourage early ANC.

We should ask a woman to have regular antenatal check-ups, through which she will also stay updated about her date. According to me if a woman is regular in her ANC check-ups she will not face any complication during delivery period. (IP06, F, MO)

Six healthcare providers called for improved availability and uptake of ultrasound scans. Four wanted additional training for frontline workers on gestational age estimation, and one (ANC09, M, MO) specifically asked to be trained on how to properly assess fundal height. While some providers said that the forms used to document ANC were easy and required no changes, one medical officer said that the ANC cards (Mamta cards) are poorly designed. Three providers expressed excitement about moving to digital record keeping, suggesting that this would reduce data entry errors. Several medical officers suggested that the oversight and training of ANMs needs be strengthened, including ensuring that ANMs complete ANC records accurately (IP05, M, MO) and that ultrasound technicians are sufficiently skilled.

## Discussion

Health care providers in rural Rajasthan reported determining gestational age and estimating date of delivery using some combination of last menstrual period, fundal height, and ultrasound. Their description of their practices showed a lack of standard protocol, varying levels of confidence in their capacity to make accurate estimates, and differing strategies for managing inconsistencies between estimates derived from different methods. We found that many frontline healthcare providers valued gestational age estimation because it could help women prepare for childbirth, but not as a tool for clinical decision making. Providers also tended to report overconfidence in the accuracy of their gestational age estimations, with only a few discussing the limitations inherent to the methods or the potential inaccuracies introduced by features of their work environment.

Providers faced a number of barriers to making accurate estimates. Accurate ultrasound-based dating was unlikely because many women received their first ANC in the second trimester and because ultrasounds required women to travel long distances, pay (in the private sector) or wait for many hours (in the public sector) and were considered unnecessary by most women and providers in normal healthy pregnancies. Health care providers considered last menstrual period to be an affordable and accessible method of estimating gestational age during pregnancy, but were limited by low client recall. In addition, providers themselves tended to assume that all women had regular menstruation prior to becoming pregnant. While fundal height assessment was popular – and was the only method that healthcare providers could themselves control completely –providers used their hands rather than measuring tapes to generate estimates rounded to months rather than weeks gestation.

Our research enabled the development a framework to understand factors that influence the accuracy, use and documentation of gestational age estimates, which identifies influences across four levels: health system, provider characteristics, client characteristics, and estimation method (Fig. 1). This framework can help policymakers, academics, and healthcare workers identify areas for intervention, as discussed below. Furthermore, this framework could help stakeholders consider the holistic environment in which gestational age estimation occurs, which will in turn enable them to identify weakness and understand how the spheres interact to build a culture around gestational age estimation, documentation and use.

Health system policymakers can consider interventions to improve gestational age estimation at multiple entry points of this framework (Table 2). Many of these interventions could be integrated within India’s National Health Mission, including its guidelines and norms for government health care facilities and staff [20, 21] and the Janani Surakha Yojana conditional cash transfer program, which is associated with more women receiving ANC and more providers conducting abdominal examinations during ANC [22].

**Table 2:** Potential entry points for improving gestational age estimation

Level	Potential intervention points to improve gestational age estimation, documentation, and use
Health system	• Clarify **protocols** and expectations on which gestational age estimation methods to use and when, how to navigate conflicting estimates, and how to document and use gestational age • Increase health system **focus on the risks of preterm birth**, and the importance of identifying preterm births quickly in order to provide supportive interventions • Build health system **capacity to use gestational age estimates** for clinical decision making, to ensure that gestational age accuracy is a meaningful part of antenatal and intrapartum care provision (i.e., ensure that interventions are actually available to address problems identified through gestational age estimation) • **Ongoing quality improvement:** Train, support, and supervise health workers on gestational age estimation, including training that compares provider estimates to a gold standard and offers feedback • Reduce provider **workload** so that they have sufficient time to assess and document gestational age during ANC • Increase the **accessibility of equipment** that aids gestational age estimation, particularly ultrasound but also measuring tapes for fundal height assessment and gestational age wheels
Frontline health care provider characteristics	• Improve providers’ **technical understanding** of gestational age estimation methods, including the inherent limitations of each method and the standardized protocols (such as ACOG [19]) for resolving discrepancies between LMP and ultrasound dating • Improve providers’ **capacity** to correctly estimate gestational age, including on how to work with women to get a best-possible last menstrual period date when possible, clarifying that estimation is from the *first day* of the last menstrual period, training on how to use a measuring tape to assess fundal height and knowing the time period within which ultrasound dating is accurate • Enable providers to build **rapport and trust with clients and communities** to encourage timely care-seeking and open discussion of menstruation and sexual activity • Providers will **value** gestational age estimates and be willing to invest time and energy into improving their accuracy and documentation when they know that gestational age estimates are important to client health • Provide **respectful and high-quality maternal healthcare** to increase the value of early and regular ANC for women
Patient characteristics	• Encourage women to seek **first ANC** as early as possible through outreach, awareness raising, and incentives since this will improve LMP recall for those with regular menstruation prior to pregnancy and will enable early ultrasound when gestational age estimation is most accurate • Build health system capacity to communicate effectively across **language** groups and among **migrants** through translation, appropriate within-community outreach efforts, and recruiting and training health care providers from linguistically diverse communities • Increase **family support** for early ANC and ultrasound by reducing financial and geographical barriers to access and communicating the value of accurate gestational age estimates • In addition to broadly increasing **women’s access to education**, engage lower-literacy women through audio visual media, community outreach and social groups to build understanding of menstruation and maternal healthcare • Equip frontline health care providers with strategies to interpret dates reported by women with different **cognitive approaches to time**, such as by linking harvests and festivals to the Gregorian calendar
Gestational age estimation method	• Interventions to improve gestational age accuracy must account for the **inherent limitations** in terms of their accuracy and the stage in pregnancy when they can be used • **Ease of use** and **equipment required** must be considered when considering strategies to improve gestational age estimation

Our study points to the need for improved access to ultrasound for gestational age estimation coupled with improving demand generation for early ANC, since so few women confidently recalled their LMP and since fundal height enables only approximate dating. However, there are tradeoffs and potential drawbacks to the increased use of ultrasound in low resource health systems [23]. Access to ultrasound only contributes to more accurate gestational age estimates if the machines produce sufficiently high quality images, which is not the case for many portable machines [23], and if providers are appropriately skilled and only use ultrasound for gestational age estimation within the recommended time period, which has been found to often not be adhered to [24]. More broadly, a randomized controlled trial in Pakistan, Kenya, Zambia, Democratic Republic of Congo and Guatemala found that increased access to ultrasound was not linked to improved client outcomes.

Given the challenges with widespread early access to ultrasound, gestational age estimation using last menstrual period remains a viable option for frontline health workers in low resource settings. However, this method yields accurate estimates only among women who had regular menstruation prior to pregnancy, something frontline healthcare providers may not account for. The proportion of pregnant women in low resource setting who have irregular or absent periods prior to pregnancy, which proscribes the use of LMP for gestational age estimation, is likely high due to undernutrition, hormonal disorders, early menopause, becoming pregnant again before postpartum menses regulates, and the use of many forms of contraception. Even among women who were menstruating regularly before becoming pregnant, communication about LMP is hindered by low client recall, poor client-provider rapport [25], shame around menstruation [26, 27], and traditional approaches to timekeeping (harvests, festivals). Efforts to reduce shame and improve women’s reproductive health knowledge may indirectly improve the use of LMP for gestational age estimation.

Late ANC reduces the likelihood that a woman will accurately recall the date of her last menstrual period and limits the capacity for ultrasound to produce an accurate gestational age estimate. ANC after the first trimester is common in many low and middle income countries, particularly among rural areas and among less educated women [28–30]. Cultural norms around shame or bad omens influence when the news of a pregnancy can be shared with one’s community: first ANC is delayed among women who wait until pregnancy is visible before talking to others, including health workers, often linked to an understanding that early pregnancy is a period of vulnerability [31, 32]. Women in South Africa [33] and India [34] have explained that they consider pregnancy a natural process with little risk to one’s health (as opposed to labour, which has clear health risks), and thus does not require multiple or early ANC. Interventions have been moderately successful at changing these norms to increase ANC uptake through participatory community groups [35], as well as media campaigns, financial incentives, and home visits by healthcare providers [36].

This study provides a strong qualitative exploration of how healthcare providers working in rural government facilities or those with knowledge of the those facilities report their practices and experiences with gestational age estimation, documentation and use. Future research that explores client perspectives would provide valuable triangulation. Moreover, additional mixed methods research could better understand associations between specific provider characteristics (such as years of experience, cadre type, whether they are from the same region as their clients or not, gender, and work environment including facility type, client load, and staffing levels) and their attitudes and approaches to gestational age estimation. Finally, although ultrasound is closely regulated in India through the 2003 Pre-Conception and Pre-Natal Diagnostic Techniques (Prohibition Of Sex Selection) Act [37] this study did not explore the potential link between regulation and availability. Future research is needed to understand whether efforts to curtail fetal sex determination have contributed to poor ultrasound access.

## Conclusions

Frontline health care providers in Rajasthan’s rural government facilities estimate gestational age using the methods most available to them and their clients. However, they receive little to no feedback on the accuracy of these assessments. Moreover, the gestational age estimates that they generate and document are not uniformly used to inform time-sensitive interventions. The limitations inherent to the estimation methods, and women’s difficulty in recalling their last menstrual period, limit gestational age estimation to the nearest month rather than week. Despite the widespread use of time-sensitive interventions including antenatal corticosteroids, providers consider the existing processes to be sufficient. Interventions to improve the accuracy of gestational age estimation should consider factors at four levels: health system, frontline provider, clients, and the gestational age methods themselves.

## Supplementary information

**Additional file 1.** Annexure 1

## Data Availability

The datasets generated and analysed during the current study are not publicly available due the risk of identifying details of the respondents being gleaned through close reading of complete transcripts of the interviews. Relevant sections of the data, such as complete code reports and large portions of transcripts, are available from the corresponding author on reasonable request.

## Abbreviations

ANC: Antenatal care
ANM: Auxiliary nurse midwife
ASHA: Accredited social health activist
EDD: Estimated date of delivery
IP: Intrapartum care
KI: Key informant
LMICs: Low- and middle-income countries
LMP: Last menstrual period
MBBS: Bachelor of Medicine, Bachelor of Surgery
MO: Medical officer
MoHFW: Ministry of Health and Family Welfare
