# Supplementary material for: “I can guess the month … but beyond that, I can’t tell” an exploratory qualitative study of health care provider perspectives on gestational age estimation in Rajasthan, India
Source: BMC Pregnancy Childbirth. 2020 Sep 11;20:529. doi: 10.1186/s12884-020-03201-6 (PMC7488485; doi:10.1186/s12884-020-03201-6)
Supplement: Supplementary file 1 — Additional file 1. Annexure 1 [file 12884_2020_3201_MOESM1_ESM.zip › annex 1 interview guide ENGLISHR3.docx]

Respondent group: Skilled **antenatal care** providers (nurses, nurse-midwives, clinical officers, and physicians)

*Thank you for speaking with me today. I am here to learn from you about gestational age estimation in your workplace. I am interested in hearing about typical practice, more so than ideal behavior. Please feel free to speak frankly about positive practices, as well as those that could be improved.*

| Domain/topic | Questions and probes |
| --- | --- |
| Opening | Please tell me a bit about yourself and your work.  *If they do not volunteer this information, probe to find out:*   - Where you are from? - How long you have been working here? - What is your current role? - How did you come to this position (e.g. career evolution, previous jobs or training…) |
| Overview of antenatal care | Could you tell me about the procedure of a typical ANC visit?  What is your role in ANC?  What are the components and priorities of ANC?   - Probe on: providing services, screening, and counseling |
| GA estimation: overview | Could you explain how you typically estimate gestational age? What method(s) are used? What methods are most important? What methods do you think are most reliable?  In an ideal world, what methods would you like to see used for gestational age estimation? What are some reasons that this ideal is not always possible?  When do you use these different methods? |
| GA estimation: last menstrual period | If they use the last menstrual period method, ask: How often are women able to reliably report the date of the first day of the last menstrual period?  What factors may make this more or less likely? [Which women are more likely to be able to report LMP? Which women are less likely to be able to report LMP?]  What kinds of techniques or prompts are used to obtain this information?  How feasible would it be to get better estimation? [Probe: What are some reasons for this?] |
| GA estimation: estimated date of conception | If they use estimated date of conception, ask: how often is this method used?  What factors affect the collection of this information, such as the timing of the first ANC visit? |
| GA estimation: fundal height & bimanual exam | What’s the standard practice related to measuring fundal height or doing a bimanual exam to estimate gestational age?  When is this typically done? (discuss both types of examination)  What are factors that affect this practice?  IF ASKED TO CLARIFY: Examples might include lack or equipment or providers’ lack of knowledge, women’s or provider’s discomfort with pelvic exam, etc.  What happens if assessment based on menstrual criteria does not match assessment based on physical examination? |
| GA estimation: ultrasound | How widely used is ultrasound in this facility [or geographic area] for routine care during pregnancy? During the first trimester?   - In what circumstances is ultrasound used? - In what circumstances is endovaginal ultrasound performed?   - Is it commonly performed?   - If not commonly performed, why not? - Where is ultrasound provided? Is it in this facility, or is it obtained from a different service provider?   - How does this differ in the public vs. the private sector? - Are practices related to ultrasound use changing? (For example, is it becoming more or less available to women from different socioeconomic levels or regions of the country?) - What is your opinion of current practice around ultrasound, such as the quality of care, cost, availability, etc. [Probe for all of these factors.] - Have you been trained on when it is appropriate or inappropriate to change a patient’s estimated date of delivery? What did you learn about this? |
| ANC environment | Where are ANC services provided? (e.g., out in the village health post/health sub center/in a specific room of the clinic?) What about gestational age estimation specifically – where does that take place? How do you feel about the location?  Could you tell me about any challenges with privacy or not having an appropriate place for history taking or physical exam? (i.e., is there a place to sit? Have the patient lie down? Wash hands?) |
| Time available | When conducting ANC, are there times when you feel rushed, like there isn’t enough time? Could you tell me about that? How does the availability of time or the number of other patients influence gestational age estimation specifically? |
| Documentation | What documents are filled out during ANC?  Tell me about the specific documents you complete for gestational age, including LMP, uterine size, estimated date of delivery, etc.  How do you feel about the completeness and accuracy of these records?  Sometimes, in other faculties, records are not complete or accurate. What could be some reasons for this?  Is filling documents an easy part of your job or sometimes difficult?  What are some issues health workers might face around filling these documents?   - Shortage of time? Not having the required document? Not having writing space or writing instruments? Documents getting wet or lost?   After filling documents, what happens to the papers? Who keeps the documents or how are they stored? Who looks at the documents?  How does this information inform health providers who see the patient later in her pregnancy? How you pass this information to the provider and what they do it? |
| Tools and equipment | What tools or equipment do you have available to you for gestational age estimation? Have you faced any challenges with not having the tools and materials that you need? Can you tell me about it? |
| Other colleagues | Are there other health workers who help you during gestational age estimation? What roles do each of you play?  Have you ever received help or advice about making gestational age estimates? Could you tell me about this?  Have you ever received training, refreshers or skills upgrades on how to estimate gestational age? Could you tell me about this? How do you feel about this?  Do health workers ever face reprimands or get scolded by supervisors because of anything related gestational age estimations? What happens? |
| Personal experiences | Could you tell me about a time when making a gestational age estimation was difficult or stressful?   - What happened? - How often is it like this?   Could you tell me about a time when making a gestational age estimation was very positive or easy?   - What happened? - How often is it like this? |
| Social relations with community members | With gestational age estimations, sometimes women may feel uncomfortable speaking about personal issues. Have you ever encountered shyness or difficulty speaking to women about certain questions? What are some sensitive questions? Which women tend to be most difficult to speak to about topics important for gestational age estimation? (E.g. caste, religious groups, unmarried women)  What are some reasons women come for ANC late? Which women are most likely to come for ANC late? (E.g. migrants, women with many other children, the very poor)  What might be some taboos or rules that pregnant women or their families follow, which affects ANC?  How comfortable do the women seem in ANC in general? During gestational age estimation? Which procedures for gestational age estimation are preferred by women? How does this affect gestational age estimation? |
| Utility of gestational age estimates | What do you think are some reasons why gestational age estimates are made?  How important is gestational age estimation to maternal and child healthcare? Why? [Probe to understand whether the provider sees gestational age estimation as something useful, actionable or important] |
| Future directions | What would help to improve the accuracy of gestational age estimation during pregnancy?  Probe on:   - Training that would help improve gestational age estimates - Equipment and staff that would help improve gestational age estimates   What could help improve the documentation of gestational age estimates? |
