# Supplementary material for: “I can guess the month … but beyond that, I can’t tell” an exploratory qualitative study of health care provider perspectives on gestational age estimation in Rajasthan, India
Source: BMC Pregnancy Childbirth. 2020 Sep 11;20:529. doi: 10.1186/s12884-020-03201-6 (PMC7488485; doi:10.1186/s12884-020-03201-6)
Supplement: Supplementary file 1 — Additional file 1. Annexure 1 [file 12884_2020_3201_MOESM1_ESM.zip › annex 1 interview guide HINDIR3.docx]

*उत्तरदाता का समूह : कौशल प्रदाता नर्स, नर्स मिडवाइव्स, क्लिनिकल ऑफिसर्स, और डॉक्टर्स.*

*आज मुझ से बात करने के लिए आपका धन्यवाद. मैं यहाँ आपके कार्यस्थान और गर्भावस्था कि अवधि के अनुमान के बारे में जानने के लिए आया/आई हूँ.आदर्श व्यवहार के बजाये मैं सामान्य व्यवहार के बारे में जानने का इच्छुक हूँ. सकारातमक व्यवहारों/आदतों के बारे में और साथ ही जिनमें सुधार किया जा सकता है उनके बारे में खुल कर बताएं.*

| विषय | प्रश्न और प्रोब्स |
| --- | --- |
| शुरुआत | कृपया मुझे आपके बारे में और आपके कार्य के बारे में थोडा सा बताएं  *यदि वे अपने आप स्वेच्छा से नहीं बताएं तो पता करने के लिए प्रोब करें.*   - आप कहाँ से है. - आप कितने समय से यहाँ पर कार्य/प्रैक्टिस कर रहे हैं. - आपकी वर्तमान भूमिका/ कार्य क्या है. - आप इस पद पर कैसे आये ( यानी कि कार्य / कैरियर कि शुरुआत, पिछला जॉब या प्रशिक्षण, क्या आपका ट्रान्सफर हुआ था आदि.) |
| *प्रसवपूर्व देखभाल काओवरव्यू* | कृपया मुझे ANC क्लिनिक पर एक सामान्य ANC दौरे/विजिट के बारे में बताएं.  ANC, में आपकी भूमिका क्या है.  ANC के घटक और प्राथमिकतायें क्या है?   - सेवा प्रदान करने, स्क्रीनिंग/जांच तथा परामर्श : के लिए प्रोब करे. |
| गर्भावस्था समयावधी का अनुमान : ओवरव्यू | कृपया बताएं आप सामान्य तौर पर गर्भावस्था की अवधी का अनुमान कैसे लगाते है? ( कौन सी विधी विधियाँ ) इस्तेमाल किये जाते हैं? कौन सी विधियाँ सबसे महत्वपूर्ण हैं? कौन सी विधियाँ आप सोचतें हैं कि सबसे भरोसेमंद हैं?    एक आदर्श माहोल में, आप कोंनसी विधि इस्तेमाल करते हुए देखना चाहती है? इसके क्या कारण है की हमेशा सही/आदर्श तरीके सम्भव नहीं हो पाते?  आप इन विभिन्न तरीको को कब प्रयोग में लाती है? |
| गर्भावस्था समयावधी का अनुमान : पिछले पीरियड से | यदि एल ऍम पी विधि का प्रयोग करती है तो पूछें, महिलाएं पहले दिन की तारीख को कब कब भरोसे से, बता पाती हैं?  ( कोनसी महिलाए एल कौन से कारक इसकी सम्भावना को कम या ज्यादा बनाते हैं?  ऍम पी की तारीख ठीक से बता पाती? कोनसी महिलाये एल ऍम पी की तारीख ठीक से नहीं बता पाती?  इस जानकारी को हासिल करने के लिए किस प्रकार की तकनीक या उत्प्रेरक/प्रोम्प्ट्स/विधियाँ इस्तेमाल की जाती हैं.  बेहतर अनुमान प्राप्त करना कितना संभव होगा ? प्रोब : इसके कारण क्या है ? |
| गर्भावस्था समयावधी का अनुमान : गर्भधारण करने कि तिथि का अनुमान | यदि वे अनुमानित तारीख की अवधारणा इस्तेमाल करती हैं तो पूछें यह विधी कब कब **इस्तेमाल** की जाती है ?  कौन से दूसरे कारक इस जानकरी के एकत्र करने को प्रभावित करते हैं, जैसे कि पहले/प्रथमANCदौरे की टाइमिंग (का समय). |
| गर्भावस्था समयावधी का अनुमान : फंडल हाइट | गर्भकाल में फंडल हाइट ( प्युबिक बोन से युटेरस के टॉप तक ) या बाईमेनुअल/हाथ जांच के अनुमान के संबन्धित, मानक प्रैक्टिस / प्रक्रिया क्या है.  यह/ऐसा आमतौर पर कब किया जाता है ? ( दोनों प्रकार की जांच के बारे में चर्चा करें)  इस प्रक्रिया को कौन से कारक प्रभावित करते हैं?  यदि स्पष्टीकरण के लिए पूछा जाए: उदहारण में उपकरणों या प्रदाताओं की कमी, ज्ञान की कमी, पेलिव्क जांच में महिला या प्रदाता को असुविधा आदि  यदि प्रत्यक्ष/शारीरिक जांच के आधार पर किये गए आंकलन, मेंसुरल मानक के आधार पर किये गए आंकलन से मेल नहीं खाते हैं तो क्या होता है. |
| गर्भावस्था समयावधी का अनुमान : अल्ट्रासाउंड | इस सुविधा ( भौगोलिक एरिया ) में **गर्भावस्था** की नियमित जांच के लिए अल्ट्रासाउंड कितनी व्यापकता से उपयोग की जाती है? पहली तिमाही के दौरान ?   - किन परिस्थितियों में अल्ट्रासाउंड इस्तेमाल की जाती है. - किन परिस्थितियों में इंडोवजाईनल अल्ट्रासाउंड इस्तेमाल की जाती है.   - क्या यह सामान्यरूप से इस्तेमाल की जाती है?   - यदि सामान्य रूप से इस्तेमाल नहीं की जाती है तो क्यों नहीं. - अल्ट्रासाउंड कहाँ प्रदान की जाती है ? क्या यह सुविधा/फैसिलिटी में है या यह अलग सेवा प्रदाता से प्राप्त की जाती है.   - क्या यह सार्वजानिक और निजी क्षेत्र में अलग अलग होती है ? - क्या अल्ट्रासाउंड से सम्बंधित आदतें/प्रैक्टिस बदल रही हैं. ( उदहारण के लिए, विभिन्न सामाजिक-आर्थिक स्तरों की महिलाओं के लिए यह कम या अधिक उपलब्ध हो रही है?) - अल्ट्रासाउंड की वर्तमान प्रैक्टिस के बारे में आपकी राय क्या है, जैसे कि देखभाल की क्वालिटी, लागत, उपलब्धता आदि [ इन सभी कारकों के लिए प्रोब करें ] - क्या आपको इस बात के लिए प्रशिक्षण मिला है कि महिला की प्रसूति की अनुमानित तिथि को बदलना कब उचित या अनुचित है. आपने इसके बारे में क्या सीखा. |
| ANC का माहौल | ANC सेवाएं कहाँ पर प्रदान की जाती है? ( यानी कि गाँव के बाहर स्वास्थ्य पोस्ट/ स्वास्थ्य उपकेंद्र/ क्लिनिक के विशेष कमरे में ?) विशेष रूप से गर्भावस्था की अवधी के अनुमान के बारे में क्या कहेगें – यह कहाँ की जाती है ? या स्थान/ लोकेशन के बारे में आपको क्या लगता है?  क्या आप मुझे, हिस्ट्री टेकिंग ( वृतांत लेने ) शारीरिक जांच करने में निजिता के बारे में कोई चुनौतियों के बारे में बतायेंगें ? ( यानी कि बैठने के लिए स्थान है या नहीं ? महिला/रोगी को लेटाने में ? हाथ धोने में ? |
| उपलब्ध समय | जब ANC की जाती है, तब किसी समय पर आपको भीड़-भाड़ महसूस होती है, जैसे कि आपके पास पर्याप्त समय नहीं होना?  क्या आप मुझे उस के बारे में बताएगें?  समय कि उपलब्धता या दूसरे रोगी/महिलाओं कि संख्या कैसे विशेष रूप से गर्भावस्था की अवधी के अनुमान/आंकलन को प्रभावित करती है. |
| डॉक्यूमेंटेशन/ कागजी कार्यवाही | ANC के दौरान कौन से डॉक्यूमेंट भरे जाते हैं?  डाक्यूमेंट्स को भरना आपके कार्य का एक सरल हिस्सा है या कभी कभी कठिन हो जाता है. LMP, अटेराईन/गर्भाशय के आकार, प्रसव/डिलीवरी कि अनुमानित तिथि आदि को शामिल करते हुए, गर्भावस्था की अवधी के लिए आप जो डाक्यूमेंट्स पूरे करते हैं उनके बारे में मुझे बताएं. क्या आप मुझे विशेष रूप से गर्भावस्था की अवधी के बारे में किसी डॉक्यूमेंटेशन के बारे में बता सकते हैं?  इस रिकॉर्ड की परिपूर्णता और सटीकता के बारे में आप क्या सोचते हैं ?  कभी कभी अन्य सुविधाओ में, रिकार्ड्स पुरे और सटिक नही होते इसके क्या कारण हो सकते है ?  डाक्यूमेंट्स को भरना आपके कार्य का एक सरल हिस्सा है या कभी कभी कठिन हो जाता है.    इन डाक्यूमेंट्स को भरने में स्वास्थ्य कार्यकर्ताओं को जिन समस्याओं का सामना करना पड़ सकता हैं वे समस्याएं क्या हैं.   - आवश्यक डॉक्यूमेंट का नहीं होना? लिखने के लिए स्थान नहीं होना या लिखे के लिए उपकरण/इंस्ट्रूमेंट नहीं होना?   डाक्यूमेंट्स भरने के बाद, कागज/पेपर्स का क्या होता है? डाक्यूमेंट्स को कौन संभालता है या वे कैसे स्टोर किये( संभाल कर रखे ) जाते हैं. डाक्यूमेंट्स को कौन देखता है?  महिला की गर्भावस्था में जो स्वास्थ्य प्रदान महिला/रोगी को बाद में देखेगें उन स्वास्थ्य प्रदाताओं को यह जानकारी कैसे ( सूचना देती है) उपलब्ध करवाती है? आप यह जानकारी प्रदाता को कैसे देती है और वे उनका क्या करते है? |
| औजार और उपकरण. | गर्भावस्था की अवधी के आंकलन के लिए आपके पास कौन कौन से औजार और उपकरण उपलब्ध है? जिन औजारों और सामग्रियों कि आपको जरुरत होती है क्या उन औजारों और सामग्रियों के आपकें पास नहीं होने के कारण आपने कभी भी कोई चुनौतियों का सामना किया है? क्या आप मुझे इसके बारे में बता सकते हैं. |
| दूसरे साथी / सह कर्मी | क्या दूसरे स्वास्थ्य कार्यकर्ता है जो गर्भावस्था की अवधी के आंकलन के लिए, आपकी मदद करते हैं. आप में से प्रत्येक की क्या भूमिका होती है.  क्या आपने गर्भावस्था की अवधी के आंकलन के लिए, कभी भी मदद या सलाह प्राप्त की है. मुझे इसके बारे में बताएं?  क्या आपने गर्भावस्था की अवधी के आंकलन के लिए, कभी भी ट्रेनिंग, रिफ्रेशर या स्किल्स प्राप्त की है. मुझे इसके बारे में बताएं? आप इसके बारे में क्या महसूस करते है ?  क्या स्वास्थ्य कार्यकर्ता को कभी ऐसी समस्यों का सामना होता है कि गर्भावस्था की अवधी के आंकलन से सम्बंधित किसी भी बात के लिए सुपरवाइजर के द्वारा डांट या धमकी दी गयी या बदजबानी की गयी, ऐसे में क्या होता है. |
| निजी अनुभव | क्या आप मुझे किसी ऐसे समय के बारे में बता सकते हैं जब गर्भावस्था की अवधी का आंकलन करना कठिन या तनावभरा होता है.   - क्या क्या हुआ था ? - ऐसा कब कब होता है?   क्या आप मुझे किसी ऐसे समय के बारे में बता सकते हैं जब गर्भावस्था की अवधी का आंकलन सकारात्मक या सरल होता है.   - क्या क्या हुआ था ? - ऐसा कब कब होता है? |
| सामुदायिक सदस्यों के साथ सामाजिक संबंध | स्वास्थ्य गर्भावस्था की अवधी के आंकलन, के बारे में कभी कभी महिला निजी समस्याओं के बारे में बताने में असहज महसूस कर सकती है. क्या आपने महिला से कुछ ख़ास प्रश्नों के बारे में बात करने में कभी भी शर्म या कठिनाई का सामना किया है. कुछ संवेदनशील प्रश्न कौन से हैं. कौन सी महिलाओं से, गर्भावस्था की अवधी के आंकलन, के विषयों/टॉपिक के बारे में बात करने में कठिनाई होती होगी. ( जाति, धार्मिक समूह, अविवाहित महिलाये )  वह कोंनसे कारण है कि महिलाये ANC के लिए देर से आती है? वह कोनसी महिलाये है जो ANC के लिए देर से आती है ( उदाहरण के तोर पर: प्रवासी , जिन महिलाओं के बच्चे पहले से है, बहुत गरीब)  वे पाबंदियां या नियम कौन से होगें जो गर्भवती महिला या उसका परिवार मानता होगा, जो ANC को प्रभावित करते हों.  सामान्य रूप से ANC में महिला कैसे/कितनी सहज लगती है.गर्भावस्था की अवधी के आंकलन के दौरान ? गर्भावस्था की अवधी के आंकलन, के लिए महिला के द्वारा कौन सी प्रक्रियाएं पसंद की जाती हैं. यह गर्भावस्था की अवधी के आंकलन, को कैसे प्रभावित करती है? |
| गर्भावस्था कीअवधी के आंकलन, की उपयोगिता | आप क्या सोचते हैं कि कौन से कारण हैं जिनकी वजह से गर्भावस्था की अवधी का आंकलन, किया जाता है.  मातृत्व और शिशु स्वास्थ्य देखरेख के लिए गर्भावस्था की अवधी का आंकलन, कितना महत्वपूर्ण है? [या समझने के लिए प्रोब करने कि क्या प्रदाता गर्भावस्था की अवधी का आंकलन, को कुछ उपयोगी, कार्रवाई योग्य करने लायक या महत्वपूर्ण, मानता है या नहीं ] |
| इसे बेहतर बनाने के लिए | गर्भावस्था के दौरान, गर्भावस्था की अवधी का आंकलन की सटीकता, में सुधार के लिए किस किस बात से मदद मिलेगी.  गर्भावस्था की अवधी का आंकलन, में सुधार के लिए किस किस बात से मदद मिल सकती है. |
