# Supplementary material for: “I can guess the month … but beyond that, I can’t tell” an exploratory qualitative study of health care provider perspectives on gestational age estimation in Rajasthan, India
Source: BMC Pregnancy Childbirth. 2020 Sep 11;20:529. doi: 10.1186/s12884-020-03201-6 (PMC7488485; doi:10.1186/s12884-020-03201-6)
Supplement: Supplementary file 1 — Additional file 1. Annexure 1 [file 12884_2020_3201_MOESM1_ESM.zip › annex 1 interview guideR3.docx]

Respondent group: Skilled **antenatal care** providers (nurses, nurse-midwives, clinical officers, and physicians)

*उत्तरदाता का समूह : कौशल प्रदाता नर्स, नर्स मिडवाइव्स, क्लिनिकल ऑफिसर्स, और डॉक्टर्स.*

*Thank you for speaking with me today. I am here to learn from you about gestational age estimation in your workplace. I am interested in hearing about typical practice, more so than ideal behavior. Please feel free to speak frankly about positive practices, as well as those that could be improved.*

*आज मुझ से बात करने के लिए आपका धन्यवाद. मैं यहाँ आपके कार्यस्थान और गर्भावस्था कि अवधि के अनुमान के बारे में जानने के लिए आया/आई हूँ.आदर्श व्यवहार के बजाये मैं सामान्य व्यवहार के बारे में जानने का इच्छुक हूँ. सकारातमक व्यवहारों/आदतों के बारे में और साथ ही जिनमें सुधार किया जा सकता है उनके बारे में खुल कर बताएं.*

| Domain/topic  विषय | Questions and probes  प्रश्न और प्रोब्स |
| --- | --- |
| Opening  शुरुआत | Please tell me a bit about yourself and your work.  कृपया मुझे आपके बारे में और आपके कार्य के बारे में थोडा सा बताएं  *If they do not volunteer this information, probe to find out:*  *यदि वे अपने आप स्वेच्छा से नहीं बताएं तो पता करने के लिए प्रोब करें.*   - Where you are from? आप कहाँ से है. - How long you have been working here? आप कितने समय से यहाँ पर कार्य/प्रैक्टिस कर रहे हैं. - What is your current role? आपकी वर्तमान भूमिका/ कार्य क्या है. - How did you come to this position (e.g. career evolution, previous jobs or training…) आप इस पद पर कैसे आये ( यानी कि कार्य / कैरियर कि शुरुआत, पिछला जॉब या प्रशिक्षण, क्या आपका ट्रान्सफर हुआ था आदि.) |
| Overview of antenatal care  *प्रसवपूर्व देखभाल काओवरव्यू* | Could you tell me about the procedure of a typical ANC visit?  कृपया मुझे ANC क्लिनिक पर एक सामान्य ANC दौरे/विजिट के बारे में बताएं.  What is your role in ANC?  ANC, में आपकी भूमिका क्या है.  What are the components and priorities of ANC?  ANC के घटक और प्राथमिकतायें क्या है?   - Probe on: providing services, screening, and counseling - सेवा प्रदान करने, स्क्रीनिंग/जांच तथा परामर्श : के लिए प्रोब करे. |
| GA estimation: overview  गर्भावस्था समयावधी का अनुमान : ओवरव्यू | Could you explain how you typically estimate gestational age? What method(s) are used? What methods are most important? What methods do you think are most reliable?  कृपया बताएं आप सामान्य तौर पर गर्भावस्था की अवधी का अनुमान कैसे लगाते है? ( कौन सी विधी विधियाँ ) इस्तेमाल किये जाते हैं? कौन सी विधियाँ सबसे महत्वपूर्ण हैं? कौन सी विधियाँ आप सोचतें हैं कि सबसे भरोसेमंद हैं?  In an ideal world, what methods would you like to see used for gestational age estimation? What are some reasons that this ideal is not always possible?  एक आदर्श माहोल में, आप कोंनसी विधि इस्तेमाल करते हुए देखना चाहती है? इसके क्या कारण है की हमेशा सही/आदर्श तरीके सम्भव नहीं हो पाते?    When do you use these different methods?  आप इन विभिन्न तरीको को कब प्रयोग में लाती है? |
| GA estimation: last menstrual period  गर्भावस्था समयावधी का अनुमान : पिछले पीरियड से | If they use the last menstrual period method, ask: How often are women able to reliably report the date of the first day of the last menstrual period?  यदि एल ऍम पी विधि का प्रयोग करती है तो पूछें, महिलाएं पहले दिन की तारीख को कब कब भरोसे से, बता पाती हैं?  What factors may make this more or less likely? [Which women are more likely to be able to report LMP? Which women are less likely to be able to report LMP?] ( कोनसी महिलाए एल कौन से कारक इसकी सम्भावना को कम या ज्यादा बनाते हैं?  ऍम पी की तारीख ठीक से बता पाती? कोनसी महिलाये एल ऍम पी की तारीख ठीक से नहीं बता पाती?  What kinds of techniques or prompts are used to obtain this information? इस जानकारी को हासिल करने के लिए किस प्रकार की तकनीक या उत्प्रेरक/प्रोम्प्ट्स/विधियाँ इस्तेमाल की जाती हैं.  How feasible would it be to get better estimation? [Probe: What are some reasons for this?]  बेहतर अनुमान प्राप्त करना कितना संभव होगा ? प्रोब : इसके कारण क्या है ? |
| GA estimation: estimated date of conception गर्भावस्था समयावधी का अनुमान : गर्भधारण करने कि तिथि का अनुमान | If they use estimated date of conception, ask: how often is this method used? यदि वे अनुमानित तारीख की अवधारणा इस्तेमाल करती हैं तो पूछें यह विधी कब कब **इस्तेमाल** की जाती है ?  What factors affect the collection of this information, such as the timing of the first ANC visit? कौन से दूसरे कारक इस जानकरी के एकत्र करने को प्रभावित करते हैं, जैसे कि पहले/प्रथमANCदौरे की टाइमिंग (का समय). |
| GA estimation: fundal height & bimanual exam  गर्भावस्था समयावधी का अनुमान : फंडल हाइट | What’s the standard practice related to measuring fundal height or doing a bimanual exam to estimate gestational age? गर्भकाल में फंडल हाइट ( प्युबिक बोन से युटेरस के टॉप तक ) या बाईमेनुअल/हाथ जांच के अनुमान के संबन्धित, मानक प्रैक्टिस / प्रक्रिया क्या है.  When is this typically done? (discuss both types of examination) यह/ऐसा आमतौर पर कब किया जाता है ? ( दोनों प्रकार की जांच के बारे में चर्चा करें)  What are factors that affect this practice? इस प्रक्रिया को कौन से कारक प्रभावित करते हैं?  IF ASKED TO CLARIFY: Examples might include lack or equipment or providers’ lack of knowledge, women’s or provider’s discomfort with pelvic exam, etc. यदि स्पष्टीकरण के लिए पूछा जाए: उदहारण में उपकरणों या प्रदाताओं की कमी, ज्ञान की कमी, पेलिव्क जांच में महिला या प्रदाता को असुविधा आदि  What happens if assessment based on menstrual criteria does not match assessment based on physical examination?  यदि प्रत्यक्ष/शारीरिक जांच के आधार पर किये गए आंकलन, मेंसुरल मानक के आधार पर किये गए आंकलन से मेल नहीं खाते हैं तो क्या होता है. |
| GA estimation: ultrasound  गर्भावस्था समयावधी का अनुमान : अल्ट्रासाउंड | How widely used is ultrasound in this facility [or geographic area] for routine care during pregnancy? During the first trimester?  इस सुविधा ( भौगोलिक एरिया ) में **गर्भावस्था** की नियमित जांच के लिए अल्ट्रासाउंड कितनी व्यापकता से उपयोग की जाती है? पहली तिमाही के दौरान ?   - In what circumstances is ultrasound used? किन परिस्थितियों में अल्ट्रासाउंड इस्तेमाल की जाती है. - In what circumstances is endovaginal ultrasound performed? किन परिस्थितियों में इंडोवजाईनल अल्ट्रासाउंड इस्तेमाल की जाती है.   - Is it commonly performed? क्या यह सामान्यरूप से इस्तेमाल की जाती है?   - If not commonly performed, why not? यदि सामान्य रूप से इस्तेमाल नहीं की जाती है तो क्यों नहीं. - Where is ultrasound provided? Is it in this facility, or is it obtained from a different service provider? अल्ट्रासाउंड कहाँ प्रदान की जाती है ? क्या यह सुविधा/फैसिलिटी में है या यह अलग सेवा प्रदाता से प्राप्त की जाती है.   - How does this differ in the public vs. the private sector? क्या यह सार्वजानिक और निजी क्षेत्र में अलग अलग होती है ? - Are practices related to ultrasound use changing? (For example, is it becoming more or less available to women from different socioeconomic levels or regions of the country?) क्या अल्ट्रासाउंड से सम्बंधित आदतें/प्रैक्टिस बदल रही हैं. ( उदहारण के लिए, विभिन्न सामाजिक-आर्थिक स्तरों की महिलाओं के लिए यह कम या अधिक उपलब्ध हो रही है?) - What is your opinion of current practice around ultrasound, such as the quality of care, cost, availability, etc. [Probe for all of these factors.] अल्ट्रासाउंड की वर्तमान प्रैक्टिस के बारे में आपकी राय क्या है, जैसे कि देखभाल की क्वालिटी, लागत, उपलब्धता आदि [ इन सभी कारकों के लिए प्रोब करें ] - Have you been trained on when it is appropriate or inappropriate to change a patient’s estimated date of delivery? What did you learn about this? क्या आपको इस बात के लिए प्रशिक्षण मिला है कि महिला की प्रसूति की अनुमानित तिथि को बदलना कब उचित या अनुचित है. आपने इसके बारे में क्या सीखा. |
| ANC environment  ANC का माहौल | Where are ANC services provided? (e.g., out in the village health post/health sub center/in a specific room of the clinic?) What about gestational age estimation specifically – where does that take place? How do you feel about the location?  ANC सेवाएं कहाँ पर प्रदान की जाती है? ( यानी कि गाँव के बाहर स्वास्थ्य पोस्ट/ स्वास्थ्य उपकेंद्र/ क्लिनिक के विशेष कमरे में ?) विशेष रूप से गर्भावस्था की अवधी के अनुमान के बारे में क्या कहेगें – यह कहाँ की जाती है ? या स्थान/ लोकेशन के बारे में आपको क्या लगता है?  Could you tell me about any challenges with privacy or not having an appropriate place for history taking or physical exam? (i.e., is there a place to sit? Have the patient lie down? Wash hands?)  क्या आप मुझे, हिस्ट्री टेकिंग ( वृतांत लेने ) शारीरिक जांच करने में निजिता के बारे में कोई चुनौतियों के बारे में बतायेंगें ? ( यानी कि बैठने के लिए स्थान है या नहीं ? महिला/रोगी को लेटाने में ? हाथ धोने में ? |
| Time available  उपलब्ध समय | When conducting ANC, are there times when you feel rushed, like there isn’t enough time? Could you tell me about that? How does the availability of time or the number of other patients influence gestational age estimation specifically?  जब ANC की जाती है, तब किसी समय पर आपको भीड़-भाड़ महसूस होती है, जैसे कि आपके पास पर्याप्त समय नहीं होना?  क्या आप मुझे उस के बारे में बताएगें?  समय कि उपलब्धता या दूसरे रोगी/महिलाओं कि संख्या कैसे विशेष रूप से गर्भावस्था की अवधी के अनुमान/आंकलन को प्रभावित करती है. |
| Documentation  डॉक्यूमेंटेशन/ कागजी कार्यवाही | What documents are filled out during ANC? ANC के दौरान कौन से डॉक्यूमेंट भरे जाते हैं?  Tell me about the specific documents you complete for gestational age, including LMP, uterine size, estimated date of delivery, etc.  डाक्यूमेंट्स को भरना आपके कार्य का एक सरल हिस्सा है या कभी कभी कठिन हो जाता है. LMP, अटेराईन/गर्भाशय के आकार, प्रसव/डिलीवरी कि अनुमानित तिथि आदि को शामिल करते हुए, गर्भावस्था की अवधी के लिए आप जो डाक्यूमेंट्स पूरे करते हैं उनके बारे में मुझे बताएं. क्या आप मुझे विशेष रूप से गर्भावस्था की अवधी के बारे में किसी डॉक्यूमेंटेशन के बारे में बता सकते हैं?  How do you feel about the completeness and accuracy of these records?  इस रिकॉर्ड की परिपूर्णता और सटीकता के बारे में आप क्या सोचते हैं ?  Sometimes, in other faculties, records are not complete or accurate. What could be some reasons for this? कभी कभी अन्य सुविधाओ में, रिकार्ड्स पुरे और सटिक नही होते इसके क्या कारण हो सकते है ?  Is filling documents an easy part of your job or sometimes difficult? डाक्यूमेंट्स को भरना आपके कार्य का एक सरल हिस्सा है या कभी कभी कठिन हो जाता है.  What are some issues health workers might face around filling these documents?  इन डाक्यूमेंट्स को भरने में स्वास्थ्य कार्यकर्ताओं को जिन समस्याओं का सामना करना पड़ सकता हैं वे समस्याएं क्या हैं.   - Shortage of time? Not having the required document? Not having writing space or writing instruments? Documents getting wet or lost? - आवश्यक डॉक्यूमेंट का नहीं होना? लिखने के लिए स्थान नहीं होना या लिखे के लिए उपकरण/इंस्ट्रूमेंट नहीं होना?   After filling documents, what happens to the papers? Who keeps the documents or how are they stored? Who looks at the documents? डाक्यूमेंट्स भरने के बाद, कागज/पेपर्स का क्या होता है? डाक्यूमेंट्स को कौन संभालता है या वे कैसे स्टोर किये( संभाल कर रखे ) जाते हैं. डाक्यूमेंट्स को कौन देखता है?  How does this information inform health providers who see the patient later in her pregnancy? How you pass this information to the provider and what they do it?  महिला की गर्भावस्था में जो स्वास्थ्य प्रदान महिला/रोगी को बाद में देखेगें उन स्वास्थ्य प्रदाताओं को यह जानकारी कैसे ( सूचना देती है) उपलब्ध करवाती है? आप यह जानकारी प्रदाता को कैसे देती है और वे उनका क्या करते है? |
| Tools and equipment  औजार और उपकरण. | What tools or equipment do you have available to you for gestational age estimation? Have you faced any challenges with not having the tools and materials that you need? Can you tell me about it? गर्भावस्था की अवधी के आंकलन के लिए आपके पास कौन कौन से औजार और उपकरण उपलब्ध है? जिन औजारों और सामग्रियों कि आपको जरुरत होती है क्या उन औजारों और सामग्रियों के आपकें पास नहीं होने के कारण आपने कभी भी कोई चुनौतियों का सामना किया है? क्या आप मुझे इसके बारे में बता सकते हैं. |
| Other colleagues  दूसरे साथी / सह कर्मी | Are there other health workers who help you during gestational age estimation? What roles do each of you play? क्या दूसरे स्वास्थ्य कार्यकर्ता है जो गर्भावस्था की अवधी के आंकलन के लिए, आपकी मदद करते हैं. आप में से प्रत्येक की क्या भूमिका होती है.  Have you ever received help or advice about making gestational age estimates? Could you tell me about this? क्या आपने गर्भावस्था की अवधी के आंकलन के लिए, कभी भी मदद या सलाह प्राप्त की है. मुझे इसके बारे में बताएं?  Have you ever received training, refreshers or skills upgrades on how to estimate gestational age? Could you tell me about this? How do you feel about this? क्या आपने गर्भावस्था की अवधी के आंकलन के लिए, कभी भी ट्रेनिंग, रिफ्रेशर या स्किल्स प्राप्त की है. मुझे इसके बारे में बताएं? आप इसके बारे में क्या महसूस करते है ?  Do health workers ever face reprimands or get scolded by supervisors because of anything related gestational age estimations? What happens? क्या स्वास्थ्य कार्यकर्ता को कभी ऐसी समस्यों का सामना होता है कि गर्भावस्था की अवधी के आंकलन से सम्बंधित किसी भी बात के लिए सुपरवाइजर के द्वारा डांट या धमकी दी गयी या बदजबानी की गयी, ऐसे में क्या होता है. |
| Personal experiences  निजी अनुभव | Could you tell me about a time when making a gestational age estimation was difficult or stressful? क्या आप मुझे किसी ऐसे समय के बारे में बता सकते हैं जब गर्भावस्था की अवधी का आंकलन करना कठिन या तनावभरा होता है.   - What happened? क्या क्या हुआ था ? - How often is it like this? ऐसा कब कब होता है?   Could you tell me about a time when making a gestational age estimation was very positive or easy? क्या आप मुझे किसी ऐसे समय के बारे में बता सकते हैं जब गर्भावस्था की अवधी का आंकलन सकारात्मक या सरल होता है.   - What happened? क्या क्या हुआ था ? - How often is it like this? ऐसा कब कब होता है? |
| Social relations with community members  सामुदायिक सदस्यों के साथ सामाजिक संबंध | With gestational age estimations, sometimes women may feel uncomfortable speaking about personal issues. Have you ever encountered shyness or difficulty speaking to women about certain questions? What are some sensitive questions? Which women tend to be most difficult to speak to about topics important for gestational age estimation? (E.g. caste, religious groups, unmarried women)  स्वास्थ्य गर्भावस्था की अवधी के आंकलन, के बारे में कभी कभी महिला निजी समस्याओं के बारे में बताने में असहज महसूस कर सकती है. क्या आपने महिला से कुछ ख़ास प्रश्नों के बारे में बात करने में कभी भी शर्म या कठिनाई का सामना किया है. कुछ संवेदनशील प्रश्न कौन से हैं. कौन सी महिलाओं से, गर्भावस्था की अवधी के आंकलन, के विषयों/टॉपिक के बारे में बात करने में कठिनाई होती होगी. ( जाति, धार्मिक समूह, अविवाहित महिलाये )  What are some reasons women come for ANC late? Which women are most likely to come for ANC late? (E.g. migrants, women with many other children, the very poor)  वह कोंनसे कारण है कि महिलाये ANC के लिए देर से आती है? वह कोनसी महिलाये है जो ANC के लिए देर से आती है ( उदाहरण के तोर पर: प्रवासी , जिन महिलाओं के बच्चे पहले से है, बहुत गरीब)  What might be some taboos or rules that pregnant women or their families follow, which affects ANC?  वे पाबंदियां या नियम कौन से होगें जो गर्भवती महिला या उसका परिवार मानता होगा, जो ANC को प्रभावित करते हों.  How comfortable do the women seem in ANC in general? During gestational age estimation? Which procedures for gestational age estimation are preferred by women? How does this affect gestational age estimation?  सामान्य रूप से ANC में महिला कैसे/कितनी सहज लगती है.गर्भावस्था की अवधी के आंकलन के दौरान ? गर्भावस्था की अवधी के आंकलन, के लिए महिला के द्वारा कौन सी प्रक्रियाएं पसंद की जाती हैं. यह गर्भावस्था की अवधी के आंकलन, को कैसे प्रभावित करती है? |
| Utility of gestational age estimates  गर्भावस्था कीअवधी के आंकलन, की उपयोगिता | What do you think are some reasons why gestational age estimates are made?  आप क्या सोचते हैं कि कौन से कारण हैं जिनकी वजह से गर्भावस्था की अवधी का आंकलन, किया जाता है.  How important is gestational age estimation to maternal and child healthcare? Why? [Probe to understand whether the provider sees gestational age estimation as something useful, actionable or important]  मातृत्व और शिशु स्वास्थ्य देखरेख के लिए गर्भावस्था की अवधी का आंकलन, कितना महत्वपूर्ण है? [या समझने के लिए प्रोब करने कि क्या प्रदाता गर्भावस्था की अवधी का आंकलन, को कुछ उपयोगी, कार्रवाई योग्य करने लायक या महत्वपूर्ण, मानता है या नहीं ] |
| Future directions | What would help to improve the accuracy of gestational age estimation during pregnancy?  गर्भावस्था के दौरान, गर्भावस्था की अवधी का आंकलन की सटीकता, में सुधार के लिए किस किस बात से मदद मिलेगी.  Probe on:   - Training that would help improve gestational age estimates - Equipment and staff that would help improve gestational age estimates   What could help improve the documentation of gestational age estimates?  गर्भावस्था की अवधी का आंकलन, में सुधार के लिए किस किस बात से मदद मिल सकती है. |
